# Increased intracranial pressure in *NF2*‑related schwannomatosis: an underestimated danger with serious consequences

**DOI:** 10.1007/s11060-026-05624-1

**Published:** 2026-05-21

**Authors:** Antoine Keraudy, Christophe Vincent, Michael Risoud, Apolline Monfilliette, Riyad Hanafi, Matthieu Peyre, Nicolas Reyns, Michel Kalamarides, Rabih Aboukais

**Affiliations:** 1 Department of Neurosurgery, Lille University Hospital, Rue Emile Laine, Lille cedex, 59037 France; 2 Department of Otology and Neurotology, Lille University Hospital, Lille, France; 3 Department of Neurology, Lille University Hospital, Lille, France; 4 Department of Neuroradiology, Lille University Hospital, Lille, France; 5 Department of Neurosurgery, Hospital Pitie-Salpetriere, AP-HP & Sorbonne Université, Paris, F- 75103 France; 6 National Reference Center for NF2-Schwannomatosis, Paris, France

**Keywords:** NF2-related schwannomatosis, Vestibular schwannoma, Meningioma, Intracranial hypertension

## Abstract

**Background:**

Advances in the management of NF2-related schwannomatosis (NF2-SWN) have improved survival, but long-term complications such as increased intracranial pressure (IICP) remain insufficiently characterized. This study investigated the mechanisms, clinical presentation, and prognostic impact of IICP in NF2-SWN.

**Methods:**

We conducted a bicentric retrospective study including 551 NF2-SWN patients followed between 1994 and 2026 in two national reference centers. IICP was defined using combined clinical, ophthalmological, and radiological criteria, including papilledema. Mechanisms were categorized into hydrocephalus, tumor-related mass effect, and venous outflow obstruction. Volumetric analyses were performed using 3D segmentation to calculate tumor volumes and annual growth rates. Clinical manifestations of IICP were also compared according to history of posterior fossa vestibular schwannoma surgery.

**Results:**

Thirty-three patients (6.0%) developed confirmed IICP, predominantly with moderate-to-severe genetic phenotypes (83%). Mechanisms included hydrocephalus (45.5%), venous outflow obstruction (36.4%), and tumor-related mass effect (15.2%). Outcomes were poor, with visual impairment in 30%, blindness in 6%, and death in 12% of patients. Annual tumor growth rates were significantly higher in patients with IICP than in controls (median 0.41 vs 0.04 year⁻¹, p = 0.011), whereas absolute tumor volume was not significantly different. Prior vestibular schwannoma surgery was independently associated with a lower frequency of headache (OR 0.06, p = 0.0019).

**Conclusions:**

IICP in NF2-SWN is an uncommon but potentially severe complication associated with major visual morbidity and mortality. Because classical symptoms may be absent, systematic ophthalmological and radiological surveillance appears essential for early detection and management.

**Supplementary Information:**

The online version contains supplementary material available at 10.1007/s11060-026-05624-1.

## Introduction

*NF2*-related schwannomatosis (*NF2*-SWN) management has changed significantly over recent decades. Improvements in multidisciplinary care, earlier diagnosis, and systemic therapies [1] have prolonged survival and reduced the need for repeated surgeries [2]. As survival increases, long-term complications and secondary disease burdens are becoming increasingly relevant challenges [3]. 

*NF2*-SWN nevertheless remains associated with significant morbidity due to multiple tumors [4]. In this setting of high intracranial tumor burden, increased intracranial pressure (IICP) may develop through heterogeneous mechanisms. These include obstructive hydrocephalus, tumor mass effect, and venous outflow impairment related to dural sinus invasion, and IICP is a known cause of morbidity and mortality in *NF2*-SWN [5]. 

In this context, diagnosis and management of IICP are challenging in *NF2*-SWN, as tumor-related neurological and ophthalmologic deficits may mask classical symptoms [6]. Moreover, asymptomatic or paucisymptomatic forms have been reported, further increasing the risk of delayed diagnosis [7]. Despite major functional consequences, particularly when visual and auditory deficits coexist, elevated ICP remains insufficiently studied in *NF2*-SWN. To date, no large cohort has described its mechanisms or prognostic implications. Better characterization of intracranial hypertension in *NF2*-SWN may improve diagnosis and management strategies.

The aim of this study was to retrospectively analyse the mechanisms, clinical presentation, and prognostic impact of IICP in a large bicentric *NF2*-SWN cohort.

## Method

### Study characteristics and population

This bicentric retrospective study included 551 patients followed between 1994 and 2026 at La Pitié Salpêtrière Hospital (AP-HP) (center 1) and Lille University Hospital (center 2), 2 major National Reference Centers for *NF2*-SWN. All patients fulfilled the 2022 revised diagnostic criteria for *NF2*-SWN [3].

**Fig. 1:**
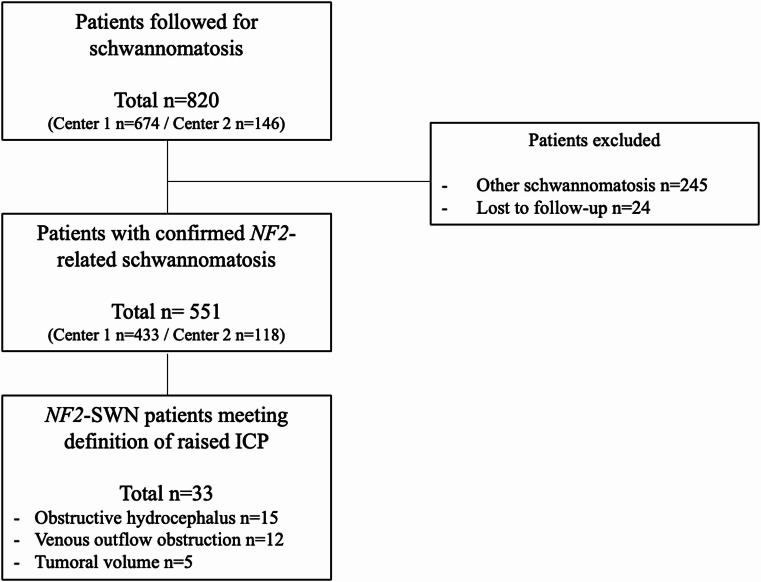
Flow-chart

Patients with other forms of schwannomatosis or those lost to follow-up defined as no clinical or imaging data for > 5 years were excluded (*n* = 269) (Fig. 1).

### Clinical, radiological and ophthalmological data

Clinical data included headache, nausea/vomiting, visual disturbances and signs of posterior fossa hypertension (neck pain, nuchal rigidity). Acute features (impaired consciousness, coma, pupillary abnormalities), timing, and onset pattern (acute, subacute < 4 weeks, chronic > 4 weeks) were recorded.

Imaging studies were reviewed to record cranial and spinal tumor locations. Tumor volumes were measured using 3D Slicer software [8]. Tumor characteristics included venous sinus involvement (Sindou classification [9]) and the presence of perilesional edema. Schwannomas were graded according to the Koos classification [10]. 

Neuro-ophthalmologic evaluations included visual acuity, fundus examination, visual field testing, and optical coherence tomography (OCT). The Genetic Severity Score for *NF2*-SWN was calculated for all patients in the raised ICP group with available genetic data [11]. 

### Raised ICP definition

Raised intracranial pressure was defined using combined ophthalmological, clinical, and radiological criteria. Isolated nonspecific symptoms such as headache, nausea/vomiting, or visual complaints without objective ophthalmological or radiological abnormalities were not considered sufficient to establish the diagnosis.

Three clinical scenarios were considered consistent with raised intracranial pressure: (1) isolated bilateral papilledema without associated clinical symptoms; (2) bilateral papilledema associated with clinical symptoms, including headache, nausea/vomiting, blurred vision, or diplopia related to sixth nerve palsy; and (3) suggestive clinical symptoms without alternative diagnosis associated with radiological findings consistent with intracranial hypertension. Radiological findings included ventricular enlargement, transependymal CSF resorption, posterior fossa compression, brain herniation, or major venous sinus involvement.

Patients underwent neuro-ophthalmological assessment including fundoscopy, visual field testing, and optical coherence tomography (OCT).

Underlying mechanisms were assessed by neuroimaging, identifying ventricular enlargement, transependymal CSF resorption, peritumoral edema, venous sinus stenosis or occlusion, and tumor-related mass effect. Patients were classified into three groups: (1) obstructive hydrocephalus, (2) tumor-related mass effect without venous impairment, and (3) tumor-associated venous outflow obstruction.

### Volumetric analysis

A subgroup analysis was performed on patients presenting with midline meningiomatosis involving venous sinus and associated intracranial hypertension that were compared with a control group with midline meningiomas invaded venous structures but without evidence of IICP. Only patients with available 3D T1-weighted gadolinium-enhanced gradient-echo MRI sequences were included in this subgroup analysis. Patients with cochlear or auditory brainstem implants (ABI), prior midline surgery, or imaging limited to spin-echo sequences were excluded in order to minimize segmentation-related artifacts.

Volumetric analysis was performed in patients with midline-invading meningiomas, comparing those with intracranial hypertension to controls with parasagittal meningiomas without ICP elevation. Tumor segmentation was conducted in 3D Slicer by a single blinded operator.

Tumor volumes were calculated by voxel-wise integration (sum of lesion voxels × voxel volume) enabling reproducible longitudinal comparisons.

Annual volumetric growth rates (*r*) were calculated using the formula:

$$\:r=\:\frac{\mathrm{ln}({V}_{t2}/{V}_{t1})}{\varDelta\:t}$$

where $\:{V}_{t1}$ and $\:{V}_{t2}$ are tumor volumes and $\:{\Delta\:}t$ is the interval in years [12].

### Management and outcomes

Clinical management included medical treatment (acetazolamide), longitudinal ophthalmological follow-up with visual acuity and fundoscopic assessment, evaluation of symptoms and papilledema regression, and implementation of palliative care when applicable. Surgical management strategies for IICP included external ventricular drainage, ventriculoperitoneal shunt placement, and tumor resection surgery, with documentation of procedure-related complications.

Patients underwent at least annual multidisciplinary follow-up organized within the reference centers, including craniospinal MRI, audiovestibular, and ophthalmological evaluations. The prognostic impact of IICP was assessed by analyzing deaths and ophthalmologic outcomes related to intracranial hypertension.

### Statistics and ethics

Continuous variables are presented as mean ± SD or median [IQR] as appropriate. Categorical variables are expressed as counts and percentages. Variables were compared using Mann–Whitney U test. Categorical variables were compared using Fisher’s exact test. To evaluate independent associations, a multivariate logistic regression model was constructed. Odds ratios (OR) with 95% confidence intervals (CI) were reported for each covariate. Model fit was assessed using the Hosmer-Lemeshow test, and discriminative performance was evaluated with the area under the receiver operating characteristic (ROC) curve (AUC). Statistical significance was set at *p* < 0.05.

The study was conducted in accordance with the Declaration of Helsinki and French regulations for retrospective research. Ethical approval received approval from the national ethics committee (IRB 00011687).

## Results

### Demographical data

The global cohort included 551 patients with *NF2*-SWN. The median age was 47.5 years (SD 18, range 3–98), and sex distribution comprised 248 men (45%) and 303 women (55%).

Thirty-three patients (6.0%) met the criteria for raised ICP, with a median age of 37.5 [IQR: 32.5–45.0], including 10 men (30.3%) and 23 women (69.7%). Among these patients, 28 (84.8%) presented with bilateral papilledema. Papilledema was isolated in 7 patients and associated with symptoms in 21, including headache in 16 and visual symptoms in 10 cases. The remaining 5 patients presented with clinical and radiological findings consistent with raised ICP, including 1 with normal fundoscopy and 4 without available fundoscopic evaluation. Among these 5 patients, 2 presented with coma associated with severe hydrocephalus or brain herniation, while 3 presented with symptomatic hydrocephalus.

Cranial and spinal lesion burden and Genetic Severity Score are reported in Table 1.

Causes of IICP included hydrocephalus in 15 patients (45.5%), venous outflow obstruction in 12 patients (36.4%), and increased tumoral volume without venous outflow obstruction in 5 patients (15.2%). Mechanism remained undetermined in 1 patient (3.0%). 

**Table 1:** Demographical data of patients with intracranial hypertension

Characteristic	
Age, median [IQR]	37.5 [32.5–45.0]
Sex	
- Men n (%) - Women n (%)	*n* = 10 (30.3%) *n* = 23 (69.7%)
Cranial lesions	
- Bilateral VS	*n = 33 (100%)*
- Non-vestibular schwannomas	*n = 6 (18.2%)*
- Meningiomas	*n = 24 (72.7%)*
• 1	*n = 3 (9.1%)*
• 2	*n = 1 (3.0%)*
• 3	*n = 3 (9.1%)*
• 4	*n = 0 (0%)*
• >5	*n = 17 (51.5%)*
Spinal lesions	
- Intradural extramedullary locations	*n* = 22 (66.7%)
- Intradural intramedullary locations	*n* = 12 (36.4%)
- Cauda equina locations	*n* = 19 (57.6%)
Cutaneous lesions	*n = 4 (12.1%)*
Genetic Severity Score	
- 1 - 2A - 2B - 3 Genetic data not-available	*n* = 1 (4%) *n* = 3 (12.5) *n* = 8 (33%) *n* = 12 (50%) *n* = 8
Criteria of raised ICP	
- Isolated bilateral papilledema - Clinical signs and bilateral papilledema- Clinical signs and radiological signs	*n* = 7 (21.2%)*n* = 21 (63.6%)*n* = 5 (15.2%)
Mechanism of intracranial hypertension	
- Hydrocephalus - Venous outflow obstruction - Tumoral volume - Unknown	*n* = 15 (45.5%) *n* = 12 (36.4%) *n* = 5 (15.2%) *n* = 1 (3.0%)

IQR = Interquartile Range

Among the 33 patients with IICP, 17 (51.5%) achieved a favorable outcome defined as resolution of intracranial hypertension-related symptoms and absence of visual sequelae, whereas 10 (30%) experienced visual loss, 2 (6%) progressed to blindness, and 4 (12%) died.

Intracranial hypertension predominantly occurred in patients with moderate to severe phenotypes, as up to half of affected patients developed permanent complications and severe sequelae.

### Clinical signs and posterior fossa surgery

**Fig. 2:**
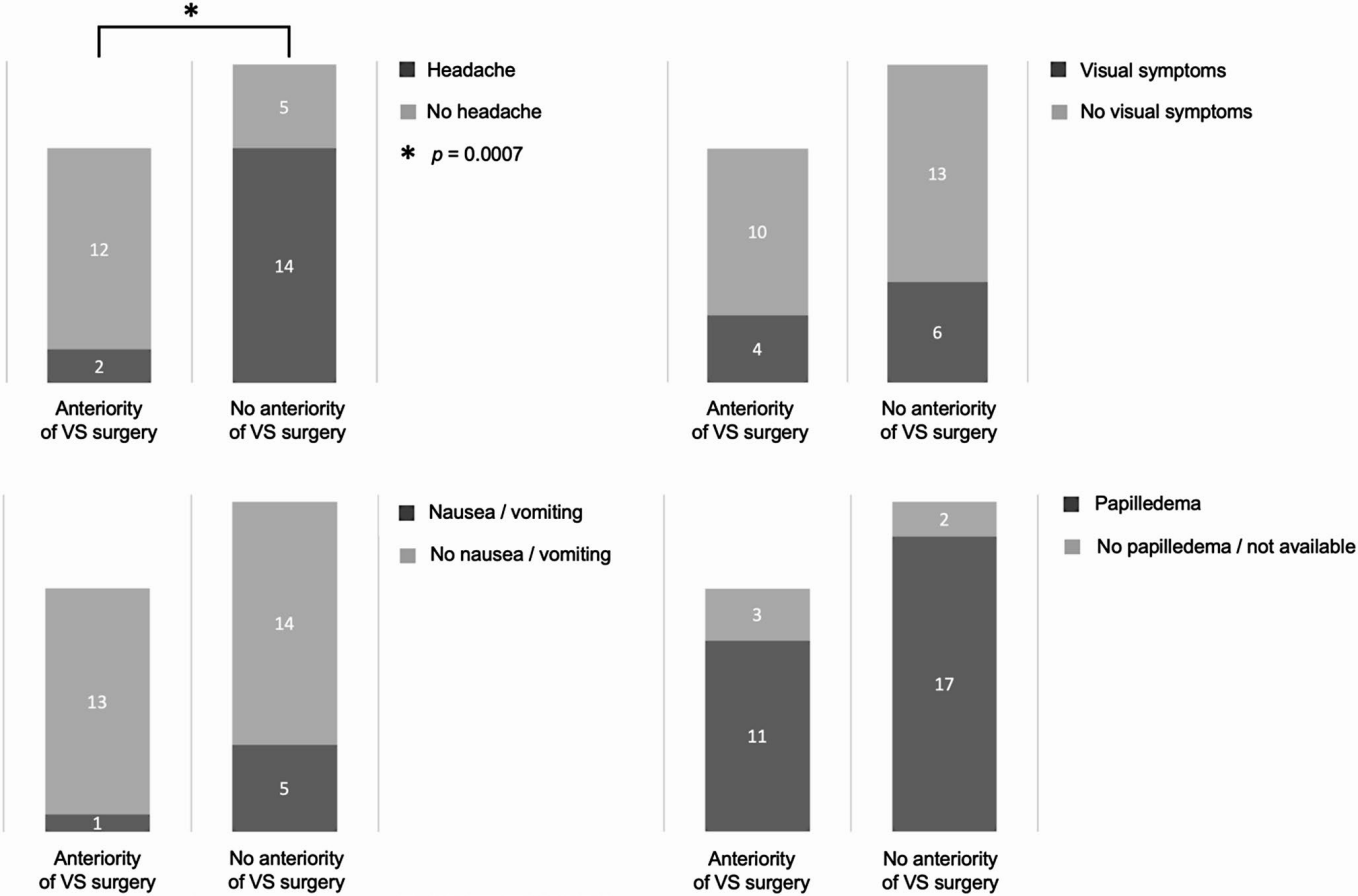
Proportion of *NF2*-SWN patients with confirmed intracranial hypertension presenting headache, visual symptoms, nausea/vomiting, and papilledema according to history of vestibular schwannoma (VS) surgery

Prior VS surgery (retrosigmoid and/or translabyrinthine approaches) was significantly associated with a lower incidence of headache in patients with intracranial hypertension. Among patients with proven intracranial hypertension and a history of VS surgery (*n* = 14), only 2 of the 14 patients (14.2%) presented with headache, compared with 14 of 19 patients (73.7%) without prior surgery (Fisher’s exact test *p* = 0.0007) (Fig. 2). The relative risk of headache in previously operated patients was 0.18 (95% CI, 0.048–0.550), corresponding to an 82% relative risk reduction, indicating lower odds of symptomatic presentation in the surgical group.

No significant association was observed between prior VS surgery and visual symptoms, which were reported in 4/14 patients (28.6%) with previous surgery versus 6/19 patients (31.6%) without previous surgery (Fisher’s exact test, *p* > 0.99). Similarly, papilledema prevalence did not significantly differ between groups, being present in 11/14 patients (78.6%) with prior surgery and 17/19 patients (89.5%) without prior surgery (Fisher’s exact test, *p* = 0.6285).

Nausea and vomiting tended to be less frequent in patients with prior VS surgery, occurring in 1/14 patients (7.1%) compared with 5/19 patients (26.3%) in the non-operated group, although this difference did not reach statistical significance (Fisher’s exact test, *p* = 0.209) (Fig. 2).

In the multivariate logistic regression model including age and sex as covariates, prior VS surgery remained independently associated with a significantly lower odds of headache (OR 0.06, 95% CI 0.005–0.38, *p* = 0.0019). The model showed good discrimination (AUC = 0.79, 95% CI 0.63–0.95, *p* = 0.005) and calibration (Hosmer-Lemeshow *p* = 0.43).

### Raised ICP groups according to mechanism

#### Hydrocephalus

Among the 15 patients with hydrocephalus, the median age was 38.0 years [31.0–51.0], with 3 men and 12 women. Genetic severity profile, clinical presentation, mechanism, and management are reported in supplementary Table 1.

Tumor locations included 8 Koos grade 4 vestibular schwannomas, 4 intraventricular meningiomas, 2 posterior fossa meningiomas, and 1 metastatic posterior fossa ependymoma with communicating hydrocephalus.

Management in the hydrocephalus subgroup primarily relied on cerebrospinal fluid diversion procedures. Ventriculoperitoneal shunting (VPS) was performed in 13/15 patients, before or after tumor resection in most cases. One patient underwent ventriculocisternostomy followed by tumor resection, and 1 was treated with acetazolamide alone.

Outcomes were favorable in 9 patients; visual loss occurred in 5 and complete blindness in 1. Among these 6 patients, 4 presented with pre-existing hearing impairment while retaining serviceable hearing, and none had undergone cochlear implantation. Two complications were reported during follow-up: extrusion of the distal catheter through the skin and a blocked ventricular catheter, both resolving without sequelae after surgery. The median follow-up after IICP diagnosis was 10 years [6.0–18.0] (Supplementary Table 1).

#### Meningioma with isolated venous obstruction

**Fig. 3:**
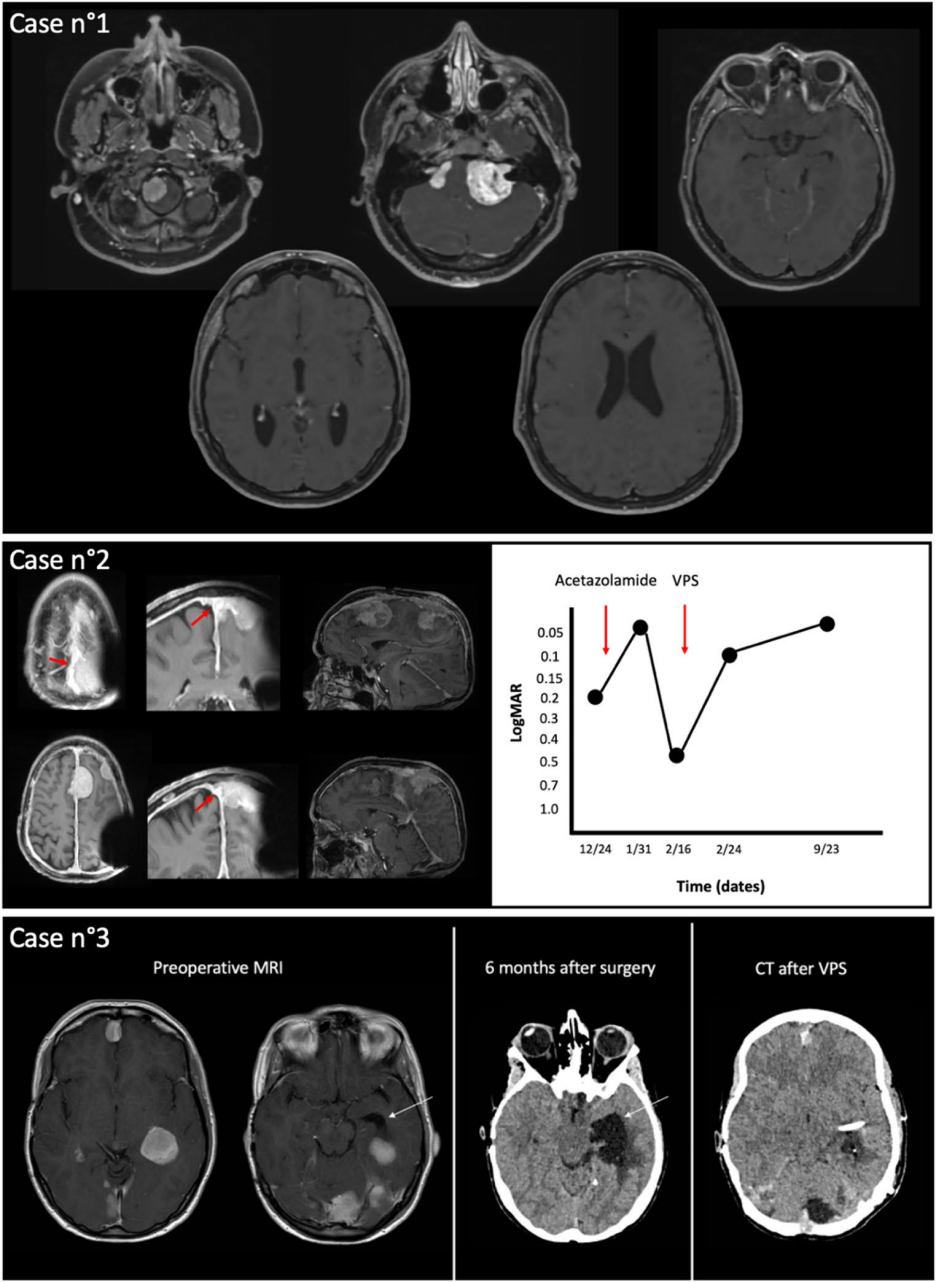
Illustrative cases of three critical scenarios: grade 4 vestibular schwannomas, midline meningiomatosis involving the superior sagittal sinus, and intraventricular meningioma. Case 1: 27-year-old with a history of isolated congenital cataract. Bilateral vestibular schwannomas and a foramen magnum meningioma were incidentally found on CT performed for cervical-brachial neuralgia. No symptoms of intracranial hypertension; bilateral grade 4 papilledema. Emergency ventriculoperitoneal (VP) shunt placed. Case 2: 70-year-old with *NF2*-SWN. Prior left VS surgery (residual facial palsy) and left-eye amblyopia. MRI showed multiple meningiomas. Developed isolated right-eye visual loss with bilateral grade 4 papilledema and tumor progression; venous outflow impairment due to superior sagittal sinus stenosis (red arrows). Acetazolamide transiently effective; VP shunt led to sustained visual improvement. (Left) T1 MRI at time of visual decline showing moderate tumor with falx and SSS involvement. (Right) Longitudinal visual acuity evolution per neuro-ophthalmology. Case 3: 46-year-old with severe *NF2*-SWN, bilateral VS previously operated. Progressive left intraventricular meningioma with obstructive hydrocephalus. Six months post-op: worsening left ventricular dilation (white arrow) with trans-ependymal resorption. No clinical signs of ICP; papilledema; visual acuity 2/10 left, 6/10 right. VP shunt performed; papilledema regressed. Patient remained shunt-dependent with multiple subsequent revisions

The venous outflow abnormality subgroup included 12 patients, with a median age of 35.5 years [28.0-39.50] (3 men, 9 women). Genetic severity scores were 2B in 5 patients, 3 in 5, and not available in 2. One of those cases is reported in (Fig. 3). 

Clinical presentation was subacute in 2 cases and chronic in 10. Headache was reported in 5 patients, visual loss in 6, and 2 patients were completely asymptomatic. Papilledema was present in all patients, with a mean duration of papilledema (time from diagnosis and regression) of 5.6 years (range 0.5–12 years) during follow-up. Mechanistically, 11 patients had parasagittal meningiomas causing superior sagittal sinus invasion, and 1 patient had a VS associated with bilateral lateral sinus stenosis.

Management strategies included tumor resection, VP shunt, diuretics (acetazolamide), venous stenting, or combination of those strategies. Among patients presenting with visual functional sequelae, 4 also had hearing impairment with preserved serviceable hearing, and 2 patients underwent cochlear implantation. The median follow-up after IICP diagnosis was 5.0 [4.0-10.50] years (Supplementary Table 2).

To explore a potential mechanistic link between venous infiltration and intracranial hypertension, we analyzed venous invasion using Sindou’s classification. Patients with IICP were compared to a control group with midline venous infiltration but no IICP. No statistically significant association was found between sinus invasion grading and the occurrence of intracranial hypertension (*p* = 0.58).

#### Tumor load without venous outflow obstruction

The tumor volume subgroup included 5 patients (3 men, 2 women). Genetic severity scores were 2 A in 2 patients, 2B in 1, and 3 in 2. Clinical presentation was acute in 3 cases and chronic in 2. Tumor locations included the convexity in 2 patients, intraventricular in 1, and posterior fossa in 2. Clinical manifestations were headache in 2 patients, altered consciousness in 2, and asymptomatic in 1. Management strategies comprised surgery in 2 patients, palliation in 2, and acetazolamide in 1. Outcomes were favorable in 3 patients and resulted in death in 2 patients. The median follow-up after IICP diagnosis was 9.0 years [3.0–10.0] (Supplementary Table 3).

### Volumetric analysis

**Fig. 4:**
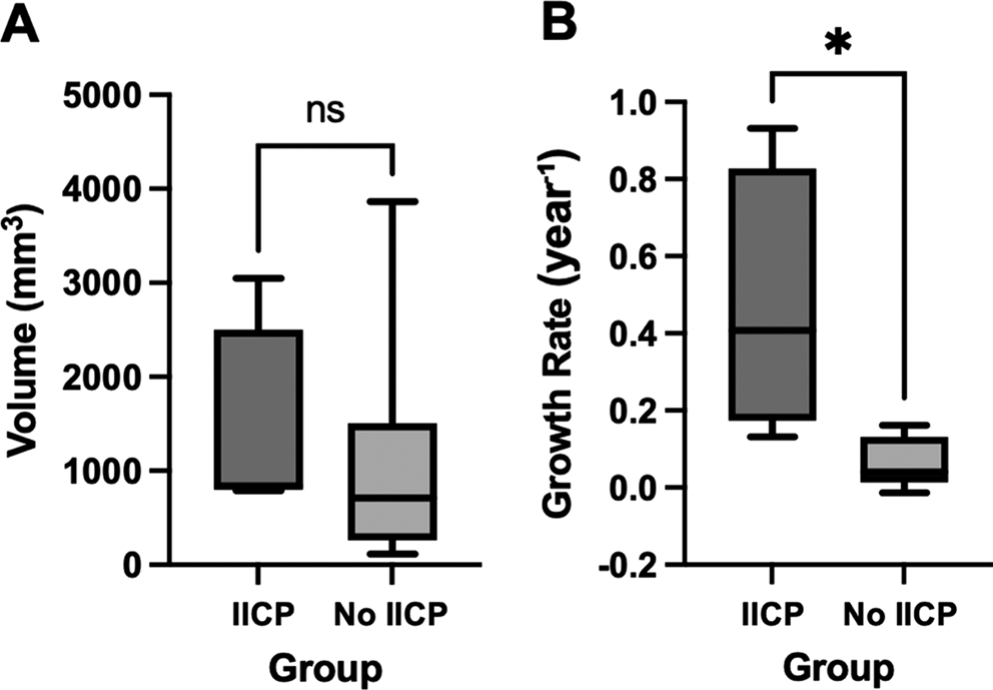
Tumor volumes and continuous annual growth rates according to increased intracranial pressure (IICP) status. (**A**) Absolute tumor volumes (mm³) in patients with IICP (*n* = 4) and without IICP (*n* = 9). (**B**) Continuous annual growth rates (r, year⁻¹) in patients with IICP and without IICP. * *p* = 0.0112

Total tumor volumes did not differ significantly between patients with IICP (IICP group, *n* = 4; median 841.5 mm³) and those without (No IICP group, *n* = 9; median 710.0 mm³; *p* = 0.2601) (Fig. 4).

Continuous annual tumor growth rates were significantly higher in patients with intracranial hypertension (median 0.4070 year⁻¹) than in those without (median 0.0403 year⁻¹; *p* = 0.0112), with a Hodges–Lehmann median difference of -0.333 year⁻¹ (No IICP Group - IICP Group), indicating faster growth in the intracranial hypertension group.

## Discussion

Raised ICP is a multifactorial complication in NF2-SWN, affecting 6% of patients in this cohort, consistent with prior reports (7% bilateral papilledema) [6]. Patients who developed intracranial hypertension were disproportionately represented in the moderate and high genetic severity categories, with 83% of affected individuals classified as moderate (2B) or severe (3). In contrast, previous literature reports that moderate and severe genotypes are less frequent, accounting for approximately 35–40% of patients [11]. This relative enrichment of higher severity genotypes suggests that IICP preferentially occurs in more severe *NF2*-SWN phenotypes.

Three tumor presentations were overrepresented among patients with intracranial hypertension: infiltrative midline meningiomas, Koos grade 4 VS with fourth ventricle obstruction, and intraventricular meningiomas, accounting for 26/33 cases (78.8%).

### Prognostic significance

Increased intracranial pressure appears to play an important role in the functional and vital prognosis during the course of the disease and may become life-threatening in fulminant cases. Across the cohort, 16 patients experienced irreversible consequences, including 11 with permanent visual loss (2 of whom were completely blind) and 5 deaths, emphasizing the critical importance of early detection and close monitoring. None of these visual deteriorations were related to direct tumor compression of the optic pathways. Among these 11 patients, 8 presented with pre-existing hearing impairment while retaining serviceable hearing, including 2 with cochlear implants. In advanced disease, predefined management strategies for acute deterioration are warranted. Visual prognosis is critical, with 38% of patients developing irreversible visual sequelae, often compounded by coexisting auditory and other ophthalmologic impairments leading to severe communication disability.

### Tumor volumes and growth dynamics

Tumor volumes increased faster in patients with raised ICP compared to those without. Median annual volumetric growth was approximately 50% annual continuous growth in the ICP group versus 4% per year in the non-ICP group, despite no significant difference in absolute tumor volumes and degree of venous sinus infiltration between groups. This suggests that rapid volumetric expansion, rather than absolute size, drives ICP elevation [13]. Rapid growth may overwhelm compensatory and venous collateralization mechanisms, predisposing to IICP even without overt hydrocephalus, as previously described [7].

Clinically, these observations support the use of segmented volumetric analyses for follow-up, with a tumor growth exceeding roughly 30% per year serving as a potential early warning signal. In such cases, closer radiologic surveillance and systematic evaluation for occult ICP elevation may allow timely intervention before clinical deterioration. This exploratory threshold may help guide surveillance in *NF2*-SWN patients. Future studies in larger cohorts are needed to validate this cutoff. Moreover, detailed assessment of midline meningioma growth patterns is warranted, as nodular growth into the sinus may pose a higher risk than outward-directed nodules that do not exacerbate venous stenosis [14]. 

### Clinical manifestations of IICP after VS surgery

In this cohort, headache appeared less frequent in patients with intracranial hypertension who had previously undergone vestibular schwannoma surgery through posterior fossa approaches. Prior surgery was associated with a significantly lower frequency of headache, whereas no significant differences were observed for visual symptoms or papilledema, and only a nonsignificant trend was observed for nausea and vomiting. These findings suggest that the clinical presentation of IICP may differ in previously operated patients. The underlying mechanism remains unclear but may relate to altered cerebrospinal fluid dynamics and changes in posterior fossa compliance following surgical opening, potentially resulting in differences in symptom latency and clinical presentation. In this context, visual symptoms related to chronic intracranial pressure elevation may occur before headache in some patients. Future studies could investigate intracranial volume distribution using advanced imaging and AI-based modeling to study longitudinal changes and correlations with clinical symptoms [15, 16]. These findings also suggest that traditional clinical signs of ICP elevation may be unreliable in *NF2*-SWN, reinforcing the need for objective diagnostic tools such as ophthalmologic evaluation, or high-resolution optic nerve MR imaging [17].

### Limitations

This study has several limitations. First, the retrospective design may have led to incomplete reporting of subtle clinical signs such as minor visual changes, potentially misestimating the prevalence of IICP. Prospective studies incorporating neuropsychological testing, quality-of-life assessments, and more advanced venous imaging follow-up could provide a more detailed understanding of the impact and underlying mechanisms of IICP in *NF2*-SWN. The proposed threshold of 30% annual tumor volumetric growth is exploratory and derived from a small sample; validation in larger, prospective cohorts is required. Finally, ICP assessments were indirect, based on clinical and ophthalmological findings, which may not capture true pressure dynamics. In this context, additional tools such as lumbar infusion testing could be explored as a direct method to detect occult ICP elevation [18].

**Table:** 

**Key Pitfalls in the Management of Raised ICP in** ***NF2*** **-SWN**
**1. Do not rely on clinical symptoms alone: **Headache, nausea, or subtle visual changes may be absent, especially after posterior fossa surgery; imaging and ophthalmological screening are essential.
**2. Absolute tumor size is not sufficient: **Rapid volumetric growth, rather than absolute tumor volume, drives ICP elevation; segmented volumetric follow-up is critical.
**3. Pay attention to high-risk locations:** Intraventricular meningiomas, Koos 4 vestibular schwannomas, and infiltrative midline nodules require close surveillance.
**4. Visual surveillance is essential: **Irreversible visual loss is worsened by auditory or other ophthalmologic impairments; High-risk patients require close follow-up to enable early intervention.
**5. Plan multidisciplinary care proactively: **Predefine intervention thresholds and management strategies, ideally within reference centers with multidisciplinary teams.

## Conclusion

In *NF2*-SWN, raised intracranial pressure is uncommon but potentially devastating, with major visual morbidity and mortality. It arises through heterogeneous mechanisms, including hydrocephalus, infiltrative midline meningiomatosis with venous outflow impairment, and rapidly growing tumors. Because classical symptoms may be absent, especially after posterior fossa surgery, systematic ophthalmological and radiological surveillance, including longitudinal volumetric and venous sinus assessment, is essential for timely detection and intervention. 

## Supplementary Information

Below is the link to the electronic supplementary material.

Supplementary Material 1

## Data Availability

The datasets generated and/or analyzed during the current study are available from the corresponding author upon reasonable request.
