# Supplementary material for: Increased intracranial pressure in NF2‑related schwannomatosis: an underestimated danger with serious consequences
Source: J Neurooncol. 2026 May 21;178(1):15. doi: 10.1007/s11060-026-05624-1 (PMC13194230; doi:10.1007/s11060-026-05624-1)
Supplement: Supplementary file 1 — Supplementary Material 1 [file 11060_2026_5624_MOESM1_ESM.docx]

**Supplementary data**

| Grade | Features |
| --- | --- |
| I | Intracanalicular tumor |
| II | Tumor extends into the cerebellopontine angle but does not extend to the brainstem |
| III | Tumor occupies the cerebellopontine cistern and contacts the brainstem but does not displace it |
| IV | Tumor displaces the brainstem |

Supplementary figure 1. Koos grading for vestibular schwannomas

| **Patient n°** | **Age, sex** | **Genetic severity score** | **Mode of presentation** | **Mechanism** | **Tumor type** | **Clinical signs** | **Papillary edema** | **Radiological signs** | **Treatment** | **Outcome** | **Follow-up (years)** |
| --- | --- | --- | --- | --- | --- | --- | --- | --- | --- | --- | --- |
| 1 | 76, W | 1A | Subacute | 4th ventricle obstruction | Grade 4 VS | Headache  Cervicalgia | N/A | Ventriculomegaly | VPS followed by tumor resection | Favorable | 10 |
| 2 | 41, M | 3 | Chronic | Communicant | Metastatic ependymoma | Pseudo- meningocele | No | Ventriculomegaly  Posterior meningocele | VPS | Favorable | 10 |
| 3 | 30, W | N/A | Subacute | 4th ventricle obstruction | Bilateral grade 4 VS  CPA meningioma | Cervicalgia | Bilateral | Slit asymmetrical ventricles | VPS | Favorable | 3 |
| 4 | 36, W | 3 | Subacute | Lateral ventricle obstruction | Intraventricular meningioma | Headache | Bilateral | Excluded ventricle | Tumor resection followed by VPS (PO day 28) | Visual loss | 6 |
| 5 | 51, W | 3 | Chronic | 4th ventricle obstruction | Grade 4 VS | No symptoms | Bilateral | Ventriculomegaly | Tumor resection | Visual loss  Optic atrophy | 10 |
| 6 | 31, W | 2B | Subacute | 4th ventricle obstruction | Grade 4 VS | Headache | N/A | Ventriculomegaly  Transependymal resorption | VPS followed by tumor resection | No visual sequelae  PO complication (distal catheter extrusion) | 7 |
| 7 | 46, W | N/A | Subacute | Lateral ventricle obstruction | Intraventricular meningioma | Headache  Visual loss | Bilateral | Temporal horn enlargement  Transependymal resorption | Tumor resection followed by VPS | No visual sequelae  PO complication (catheter occlusion) | 10 |
| 8 | 69, W | N/A | Subacute | 4^th^ ventricle obstruction | Grade 4 VS recurrence | Visual loss  No headache | Bilateral | Temporal horn enlargement  Transependymal resorption | VPS | Blindness | 23 |
| 9 | 30, W | N/A | Acute | 4th ventricle obstruction | Intraventricular meningioma | Headache  Nausea / vomiting | Bilateral | Ventriculomegaly  Transependymal resorption | Tumor resection followed by VPS | Favorable | 21 |
| 10 | 35, M | N/A | Acute | 4th ventricle obstruction | Grade 4 SV | Headache | Bilateral | Ventriculomegaly  Transependymal resorption | Ventriculocisternostomy followed by tumor resection | Favorable | 18 |
| 11 | 38, W | N/A | Chronic | 3^rd^ ventricle obstruction | Intraventricular meningioma | Headache  Visual loss | Bilateral | Ventriculomegaly  Transependymal resorption | Tumor resection followed by VPS | Visual loss | 32 |
| 12 | 30, W | 3 | Chronic | 4th ventricle obstruction | Posterior fossa meningioma | No symptoms | Bilateral | Ventriculomegaly  Transependymal resorption | Tumor resection followed by VPS | No sequelae  No complication | 5 |
| 13 | 62, W | 2B | Chronic | 4^th^ ventricle obstruction | Grade 4 VS | No symptoms | Bilateral | Ventriculomegaly  Transependymal resorption | VPS followed by resection surgery | Severe visual loss | 12 |
| 14 | 39, W | 2A | Chronic | Craniovertebral junction obstruction | Craniovertebral junction meningioma | No symptoms | Bilateral | Ventriculomegaly | Acetazolamide | Favorable | 5 |
| 15 | 34, M | 3 | Subacute | 4^th^ ventricle obstruction | Grade 4 VS recurrence | Headache | Bilateral | Ventriculomegaly | VPS | Visual loss | 8 |

Supplementary table 1. Clinical, Radiological, and Therapeutic Characteristics of *NF2-*SWN Patients with Hydrocephalus

Favorable outcome = resolution of clinical symptoms and papilledema without permanent sequelae.

N/A=Not Available; W=Woman; M=Man; CPA=Cerebello-Pontine Angle; VS=Vestibular Schwannoma; VPS=Ventriculoperitoneal Shunt; PO= Post-operative

| **Patient n°** | **Age (y), sex** | **Genetic Severity Score** | **Mode of presentation** | **Mechanism of venous impairment** | **Initial clinical signs** | **Clinical course** | **Duration** (years) | **Papilledema** | **Radiological features** | **Treatment strategy** | **Final Outcome** | **Follow-up (y)** |
| --- | --- | --- | --- | --- | --- | --- | --- | --- | --- | --- | --- | --- |
| 1 | 16, W | 3 | Subacute | SSS invasion | Headache | Headache | 2 | Bilateral | Evolutive parasagittal meningioma | Tumor resection | Favorable | 6 |
| 2 | 71, M | N/A | Subacute | SSS invasion | Visual loss  No headache | Visual loss | 0,5 | Bilateral | Evolutive parasagittal meningiomas | Acetazolamide  VPS | Visual loss  Optic atrophy | 16 |
| 3 | 23, W | 3 | Chronic | SSS invasion | No symptom | No symptom | N/A | Bilateral | Evolutive parasagittal meningiomas | Acetazolamide | Favorable | 4 |
| 4 | 28, M | N/A | Chronic | Lateral sinus stenosis | Visual loss | Visual loss | 5 | Bilateral | Bilateral grade 4 VS associated with LS stenosis | Acetazolamide  Lateral sinus stenting | Visual loss  Optic atrophy | 5 |
| 5 | 40, W | 2B | Chronic | SSS invasion | Visual loss  No headache | Visual loss | 4 | Bilateral | Evolutive midline meningiomas | Acetazolamide  VPS | Favorable visual outcome  Optic atrophy | 4 |
| 6 | 36, W | 2B | Chronic | SSS invasion | Headache  No visual loss | Headache | 2 | Bilateral | Evolutive parasagittal meningiomas | Tumor resection | Favorable | 4 |
| 7 | 35, W | 2B | Chronic | SSS invasion | Headache  No visual loss | Headache  Visual loss | 7 | Bilateral | Evolutive parasagittal meningiomas | Tumor resection  VPS | Death | - |
| 8 | 28, M | 2B | Chronic | SSS invasion | Headache  Visual loss | Blindness | 10 | Bilateral | Evolutive parasagittal meningiomas | Tumor resections (x3) | Blindness | 5 |
| 9 | 36, W | 2B | Chronic | SSS invasion | Visual loss  No headache | Visual loss | 5 | Bilateral | Evolutive parasagittal meningiomas | Tumor resection | Death | - |
| 10 | 34, W | 3 | Chronic | SSS invasion | Visual loss | Visual loss | 7 | Bilateral | Parasagittal meningiomas | Acetazolamide followed by VPS  Acetazolamide restarted | Visual loss | 12 |
| 11 | 38, W | 3 | Chronic | SSS invasion | Asymptomatic | Visual loss | 12 | Bilateral | Parasagittal meningiomas | Multiples tumoral resections  Acetazolamide 🡪 VAS | Visual loss | 1 |
| 12 | 56, W | 3 | Chronic | SSS invasion | Headache | Headache | 7 | Bilateral | Parasagittal meningiomas | Multiple tumoral resections | Visual loss | 10 |

Supplementary table 2. Clinical, Radiological, and Therapeutic Characteristics of *NF2*-SWN Patients with Venous Outflow-related intracranial hypertension

Favorable outcome = resolution of clinical symptoms and papilledema without permanent sequelae.

N/A=Not Available; W=Woman; M=Man; VS=Vestibular Schwannoma; VPS=Ventriculoperitoneal Shunt; SSS=Superior sagittal sinus; LS=Lateral sinus

| **Patient n°** | **Age, sex** | **Genetic severity score** | **Mode of presentation** | **Dominant intracranial burden** | **Main location** | **Clinical signs** | **Papillary edema** | **Radiological signs** | **Treatment** | **Outcome** | **Follow-up** |
| --- | --- | --- | --- | --- | --- | --- | --- | --- | --- | --- | --- |
| 1 | 49, M | 3 | Acute | Meningioma | Convexity | Consciousness trouble | N/A | Subfalcine engagement | Palliative care | Death | - |
| 2 | 42, W | 2A | Acute | Meningioma | Intraventricular | Headache  Cognitive impairment | Bilateral | Giant intraventricular meningioma | Tumor resection | Favorable | 9 |
| 3 | 67, M | 3 | Acute | Meningioma | Posterior fossa | Consciousness trouble | N/A | Brainstem compression | Palliative care | Death | - |
| 4 | 41, M | 2A | Chronic | Meningioma | Convexity | Headache | Bilateral | Tumor with oedema | Tumor resection | Favorable | 10 |
| 5 | 35, W | 2B | Chronic | Vestibular Schwannoma | Posterior fossa | Asymptomatic | Bilateral | Bilateral VS  Slit ventricles | Acetazolamide | Favorable | 3 |

Supplementary table 3. Clinical, Radiological, and Therapeutic Characteristics of *NF2*-SWN Patients with Tumor Volume-Related Hypertension

Favorable outcome = resolution of clinical symptoms and papilledema without permanent sequelae.

N/A=Not Available; W=Woman; M=Man; VS=Vestibular Schwannoma; VPS=Ventriculoperitoneal Shunt; SSS=Superior sagittal sinus; LS=Lateral sinus
